# Supplementary material for: Heat adaptation of phage T7 under an extended genetic code
Source: Virus Evol. 2021 Dec 1;7(2):veab100. doi: 10.1093/ve/veab100 (PMC8923235; doi:10.1093/ve/veab100)
Supplement: veab100_Supp [file veab100_supp.zip › Sequencing_Analysis.zip!/Sequencing_Analysis/A1/evidence/SNP_5.html]

BRESEQ :: Evidence


| Predicted mutation | | | | | | |
| --- | --- | --- | --- | --- | --- | --- |
| evidence | seq id | position | mutation | annotation | gene | description |
| RA | NC\_001604.1 | 21,279 | C→T | intergenic (–/–) | – / – | –/– |

| Read alignment evidence... | | | | | | | | | | | |
| --- | --- | --- | --- | --- | --- | --- | --- | --- | --- | --- | --- |
|  | seq id | position | | ref | new | freq | score (cons/poly) | reads | annotation | genes | product |
| \* | NC\_001604.1 | 21,279 | 0 | C | T | 99.0% | 7010.0 / 13.4 | 3144 | intergenic (–/–) | *–/–* | –/– |
| Reads supporting (aligned to +/- strand):  **ref** base C (22/9);  **new** base T (1558/1529);  **total** (1593/1551) | | | | | | | | | | | |
| Fisher's exact test for biased strand distribution *p*-value = 2.91e-02 | | | | | | | | | | | |
| Kolmogorov-Smirnov test that lower quality scores support variant *p*-value = 9.99e-01 | | | | | | | | | | | |

Only 100 of 3145 total aligned reads displayed.  

`CTTGCCCATACATCCCGATTCGGATGGTCAGACTAGATGGTGAATCCTACGGTCGTTCGTACATTGAGGAATACTTAGGTGACTTACGGTCCCTTGAAAATCTCCAAGAGGCTATCGTCAAGATGTCCATGATTAGCTCTAAGGTTATCGGCTTAGTGAATCCTGCTGGTATCACCCAGCCACGCCGACTGACCAAAGCTCAGACTGGTGACTTCGTTACTGGTCGTCCAGAAGACATCTCGTTCCTCCAACTGGAGAAGCAAGCAGACTTTACTGTAGCTAAAGCCGTAAGTGACGCTATCGAGGCTCGCCTTTCGTTTGCCTTTATGTTGAACTCTGCGGTTCAGCGTACAGGTGAACGTGTGACCGCCGAAGAGATTCGGTATGTAGCTTCTGAACTTGAAGATACTTTAGGTGG‑TGTCTACTCTATCCTTTCTCAAGAATTACAATTGCCTCTGGTACGAGTGCTCTTGAAGCAACTACAAGCCACGCAACAGATTCCTGAGTTACCTAAGGAAGCCGTAGAGCCAACCATTAGTACAGGTCTGGAAGCAATTGGTCGAGGACAAGACCTTGATAAGCTGGAGCGGTGTGTCAC  >  NC_001604.1/20982‑21579`  

                                                                                                                                                                                                                                                                                                         `|`                                                                                                                                                                                                                                                                                                               
  `tGCCCATACATCCCGATTCGGATGGTCAGACTAGATGGTGAATCCTACGGTCGTTCGTACATTGAGGAATACTTAGGTGACTTACGGTCCCTTGAAAATCTCCAAGAGGCTATCGTCAAGATGTCCATGATTAGCTCTAAGGTTATCGGCTTAGTGAATCCTGCTGGTATCACCCAGCCACGCCGACTGACCAAAGCTCAGACTGGTGACTTCGTTACTGGTCGTCCAGAAGACATCTCGTTCCTCCAACT‑GATAAGCAAGCAGACTTTACTGTAGCTAAAGCCGTAAGTGACGTTATCGa                                                                                                                                                                                                                                                                                                         >  1:345869/1‑301 (MQ=255)`  
    `cccATACATCCCGATTCGGATGGTCAGACTAGATGGTGAATCCTACGGTCGTTCGTACATTGAGGAATACTTAGGTGACTTACGGTCCCTTGAAAATCTCCAAGAGGCTATCGTCAAGATGTCCATGATTAGCTCTAAGGTTATCGGCTTAGTGAATCCTGCTGGTATCACCCAGCCACGCCGACTGACCAAAGCTCAGACTGGTGACTTCGTTACTGGTCGTCCAGAAGACATCTCGTTCCTCCAACTGGAGAAGCAAGCAGACTTTACTGTAGCTAAAGCCGTAAGTGACGTTATCGAg                                                                                                                                                                                                                                                                                                        >  1:291542/1‑301 (MQ=255)`  
           `aTCCCGATTCGGATGGGCAGACTAGATGGTGAATGCTACGGTCGTTCGTACATTGATGAATACTAAGGTGACTCACGGTCCCTTGAAAATCTCCAAGAGGCTATCGTCAAGATGTCCATGATTAGCTCTAAGGTTATCGGCTTAGTGAATCCTGCTGGTATCACCCAGCCACGCCGACTGACCAAAGCTCAGACTGGTGACTTCGTTACTGGTCGTCCAGAAGACATCTCGTTCCTCCAACTGGAGAAGCAAGCAGACTTTACTGTAGCTAAAGCCGTAAGTGACGTTATCGAGGCTCGcc                                                                                                                                                                                                                                                                                                 <  1:220727/301‑1 (MQ=255)`  
                  `tgtggataagagacagaGAAGGTTAATCCTACGGTCGTTCATACATTGAGGAATACTTAGGTGACTTACGGCCCATTGAAAATCTCCAAGAGGCTATCGTCAAGATGTCCATGATTAGCTCTAAGGTTATCGGCTTAGTGAATCCTGCTGGTATCACCCAGCCACGCCGACTTACCAAAGCTCAGACTGGTGACTTCGTTAATGGTCGTCCAGAAGACATCTCGTTCCTCCAACTGGAGAAGCAAGCAGACTTTACTGTAGCTAAAGCCGTAAGTGACGTTATCGAGGCTCGCCTTTCGtt                                                                                                                                                                                                                                                                                          <  1:159103/285‑1 (MQ=255)`  
                      `ctcggagatgtggataagaggcagcTACGGTCGTTCGTACATTGGGGAATACTTAGGTGACTTACTGTCCCTTGAAAATCTCCAAGAGGCTATCGTCAAGATGTCCATGATTAGCTCTAAGGTTATCGGCTTAGTGAATCCTGCTGGTATCACCCAGCCACGCCGACTGACCAAAGCTCAGACTGGTGACTTCGTTACTGGTCGTCCAGAAGACATCTCGTTCCGCCAACTGGAGAAGCAAGCAGACTTTACTGTAGCTAAAGCCGTAAGTGACGTTATCGAGGCTCGCCTTTCGTTTGcc                                                                                                                                                                                                                                                                                      <  1:286827/277‑1 (MQ=255)`  
                             `ggACTAGATGGTGAATCCTACGGTCGTTCGTACATTGAGGAATACTTAGGTGACTTACGGTCCCTTGAAAATCTCAAAGAGGCTATCGTCAAGATGTCCATGATTAGCTCTAAGGTTATCGGCTTAGTGAATCCTGCTGGTATCACCCAGCCACGCCGACTGACCAAAGCTCAGACTGGTGACTTCGTTACTGGTCGTCCAGAAGACATCTAGTTCCTCCAACTGGAGAAGCAAGCAGACTTTACTGTAGCTAAAGCCGTAAGTGACGTTATCGAGGCTCGCCTTTCGTTTGCCTTTATGt                                                                                                                                                                                                                                                                               <  1:5116/300‑1 (MQ=255)`  
                             `agagacagtGGTGAATCCTACGGTCGTTCGTACATTGAGGAATACTTAGGTGACTTACGGTCCCTTGAAAATCTCCAAGAGGCTATCGTCAAGATGTCCATGATTAGCTCTAAGGTTATCGGCTTAGTGAATCCTGCTGGTATCACCCAGCCACGCCGACTGATCAAAGCTCAGACTGGTGACTTCGTTACTGGTCGTCCAGAAGACATCTCGTTCCTCCAACTGGAGAAGCAAGCAGACTTTACTGTAGCTAAAGCCGTAAGTGACGTTATCGAGGCTCGCCTTTCGTTTGCCTTTATGt                                                                                                                                                                                                                                                                               <  1:240243/293‑1 (MQ=255)`  
                             `aGACTAGATGGTGAATCCTACGGTCGTTCGTACATTGAGGAATACTTAGGTGACTTACGGTCCCTTGAAAATCTCCAAGAGGCTATCGTCAAGATGTCCATGATTAGCTCTAAGGTTATCGGCTTAGTGAATCCTGCTGGTATCACCCAGCCACGCCGACTGACCAAAGCTCAGACTGGTGACTTCGTTACTGGTCGTCCAGAAGACATCTCGTTCCTCCAACTGGAGAAGCAAGCAGACTTTACTGTAGCTAAAGCCGTAAGTGACGTTATCGAGGCTCGCCTTTCGTTTGCCTTTATGt                                                                                                                                                                                                                                                                               <  1:439047/301‑1 (MQ=255)`  
                              `gACTATATGGTGAATCCTACGGTCGTTCGTACCTTGAGGAATACTTAGGTGACTTACGGTCCCTTGAAAATCTCCAAGAGGCTATCGTCAAGATGTCCATGATTAGCTCTAAGGTTATCGGCTTAGTGAATCCTGCTGGTATCACCCAGCCACGCCGACTGACCAAAGCTCAGACTGGTGACTTCGTTACTGGTCGTCCAGAAGACATCTAGTTCCTCCAACTGGAGAAGCAAGCAGACTTTACTGTAGCTAAAGCCGTAAGTGACGTTATCGAGGCTCGCCTTTCGTTTGCCTTTATGtt                                                                                                                                                                                                                                                                              >  1:139867/1‑301 (MQ=255)`  
                              `gACTAGATGGTGAATCCTACGGTCGTTCGTACATTGAGGAATACTTAGGTGACTTACGGTCCCTTGAAAATCTCCAAGAGGCTATCGTCAAGATGTCCATGATTAGCTCTAAGGTTATCGGCTTAGTGAATCCTGCTGGTATCACCCAGCCACGCCGACTGACCAAAGCTCAGACTGGTGACTTCGTTACTGGTCGTCCAGAAGACATCTCGTTCCTCCAACTGGAGAAGCAAGCAGACTTTACTGTAGCTAAAGCCGTAAGTGACGTTATCGAGGCTCGCCTTTCGTTTGCCTTTATGtt                                                                                                                                                                                                                                                                              >  1:72341/1‑301 (MQ=255)`  
                                  `ggagatgtgtataagagacagttcgtACATTGAGGAATACTTAGGTGACTTACGGTCCCTTGAAAATCTCCAAGAGGCTATCGTCAAGATGTCCATGATTAGCTCTAAGGTTATCGGCTTAGTGAATCCTGCTGGTATCACCCAGCCACGCCGACTGACCAAAGCTCAGACTGGTGACTTCGTTACTGGTCGTCCAGAAGACATCTCGTTCCTCCAACTGGAGAAGCAAGCAGACTTTACTGTAGCTAAAGCCGTAAGTGACGTTATCGAGGCTCGCCTTTCGTTTGCCTTTATGTTGAAc                                                                                                                                                                                                                                                                          <  1:412335/281‑1 (MQ=255)`  
                                  `aGATGGTGAATCCTACGGTCGTTCGTACATTGAGGAATACTTAGGTGACTTACGGTCCCTTGAAAAACTCCAAGAGGCTATCGTCAAGATGTCCATGATTAGCTCTAAGGTTATCGGCTTAGTGAATCCGGCTGGTATCACCCAGCCACGCCGACTGACCAAAGCTCAGACTGGTGACTTCGTTACTGGTCGTCCAGAAGACATCTCGTTCCTCCAACTGGAGAAGCAAGCATACTTTACTGTAGCTAAAGCCGTAAGTGACGTTATCGAGGCTCGCCTTTCGTTTGCCTTTATGTTGAAc                                                                                                                                                                                                                                                                          <  1:183408/301‑1 (MQ=255)`  
                                    `aTGGTGAATCCTACGGTCGTTCGTCCATTTAGGAATACTTAGGTGACTTACGGTCCCTTGAAAATCTCCAAGAGGCTATCGTCAAGATGTCCATGATTAGCTCTAAGGTTATCGGCTTAGTGAATCCTGCTGGTATCACCCAGCCACGCCGACTGACCAAAGCTCAGACTTGTGACTTCGTTACTGGTCGTCCAGAAGACATCTCGTTCCTCCAACTGGAGAAGCAAGCAGACTTTACTGTAGCTAAAGCCGTAAGTGACGTTATCGAGGCTCGCCTTTCGTTTGCCTTTATGTTGAActc                                                                                                                                                                                                                                                                        <  1:277165/301‑1 (MQ=255)`  
                                    `aTGGTGAATCCTACGGTCGTTCGTACATTGAGGAATACTTAGGTGACTTACGGTCCCTTGAAAATCTCCAAGAGGCTATCGTCAAGATGTCCATGATTAGCTCTAAGGTTATCGGCTTAGTGAATCCTTCTGGTATCACCCATCCACGCCGACTTACCAAAGCTCAGACTGGTGACTTCGTTACTGGTCTTCCAGAAGACATCTCGTTCCTCCAACTGGAGAAGCAAGCAGACTTTACTGTAGCTAAAGCCGTAAGTGACGTTATCGCGGCTCGCCTTTCGTTTGCCTTTATTTTGATctt                                                                                                                                                                                                                                                                        >  1:143289/1‑300 (MQ=255)`  
                                    `aTGGGGAATCCTACGGTCGTTCGTACATTGAGGAATACTTAGGTGAAGTACGGTCCCTGGAAAATCTCCAAGAGGCTATCGTCAAGATGTCCATGATTAGCTCTAAGGTTATCGGCTTAGTGAATCCTGTTGTTATCACCCAGCCACGCCGACTGACCAAAGCTCAGACTGGTGACTTCGTTACTGGTCGTCCAGAAGAAATCGCGTTCCTCCAACTGGAGAAGCAAGCAGACTTTACTGTAGCTAAAGCCGTAAGTGACGTTATCGAGGCTCGCCTTTCGTTTGCCTTTATGTTGAActc                                                                                                                                                                                                                                                                        <  1:42089/301‑1 (MQ=255)`  
                                         `tgtgtataagagacagcgtACATTGAGGAATACTTAGGTGACTTACGGTCCCTTGAAAATCTCCAAGAGGCTATCGTCAAGATGTCCATGATTAGCTCTAGGGTTATCGGCTTAGTGAATCCTGCTGGTATCACCCAGCCACGCCGACTGACCAAAGCTCAGACTGGTGACTTCGTTACTGGTCGTCCAGAAGACATCTCGTTCCTCCAACTGGAGAAGCAAGCAGACTTTACTGTAGATAAAGCCGTAAGTGACGTTATCGAGGCTCGCCTTTCGTTTGCCTTTATGTTGAACTCTGCgg                                                                                                                                                                                                                                                                   <  1:376838/285‑1 (MQ=255)`  
                                            `cccTACGGTCGTTCGTACATTGAGGAATACTTAGGTCACTTACTGTCCCTAGAAAATCTCCAAGAGGCTATCGTCAAGATGTCCATGATTAGCGCTAAGATTATCGGCTTAGTGAATTCTGCTGGTATCACCCAGCCACGCCGACGGACCAAAGCTCAGACTGGTGACTTCGTTACTGGTCGTCCAGAAGACATCTCGTTCCTCCAACTTTAGAAGCAAGCAGACTTTACTGTAGCTAAAGCCGTAAGTGACGTTATCGAGGCTCGCCTTTCGTTTGCCTTTATGTTGAACTCTGCGGTTc                                                                                                                                                                                                                                                                <  1:192345/300‑1 (MQ=255)`  
                                                   `gTCGTTCGTACATTGAGGAATACTTAGGTGACTTACGGTCCCTTGAAAATCTCCAAGAGGCTATCGTCAAGATGTCCATGATTAGCTCTAAGGTTATCGGCTTAGTGAATCCTGCTGGTATCACCCAGCCACGCCGACTGACCAAAGCTCAGACTGGTGACTTCGTTACTGGTCGTCCAGAAGACATCTCGTTCCTCCAACTGGAGAAGCAAGCAGACTTTACTGTAGCTAAAGCCGTAAGTGACGTTATCTAGGCTCGCCTTTCGTTTGCCTTTATGTTGAACTCTGCGGTTCAGCGTAc                                                                                                                                                                                                                                                         <  1:22578/301‑1 (MQ=255)`  
                                                     `cgttcgtACATTGAGGAATACTTAGGTGACTTACGGTCCCTTGAAAATCTCCAAGAGGCTATCGTCAAGATGTCCATGATTAGCTCTAAGGTTATCGGCTTAGTGAATCCTGCTGGTATCACCAAGCCACGCCGACTGACCAAAGCTCAGACTGGTGACTTCGTTACTGGTCGTCCAGAAGACATCTCGTTCCTCCAACTGGAGAAGCCAGCAGACTTTACTGTAGCTAAAGCCGTAAGTGACGTTATCGAGGCTCGCCTTTCGTTTGCCTTTATGTTGAACTATGCGGTTCAGCGTAcct                                                                                                                                                                                                                                                       >  1:96348/1‑299 (MQ=255)`  
                                                       `ttcgtACATTGAGGAATACTTAGGTGACTTACGGTCCCTTGGAAATCTCCAAGAGGCTATCGTCAAGATGTCCATGATTAGCTCTAAGGTTATCGGCTTAGTGAATCCTGCTGGTATCACCCCGCCACGCCGACTGACCAAAGCTCAGACTGGTGACTTCGTTACTGGTCGTCCAGAAGACATCTCGTTCCTCCAACTGGAGAAGCAAGCAGACTTTACTGTAGCTAAAGCCGTAAGTGACGTTATCGAGGCTCGCCTTTCGTTTGCCTTTATGTTGAACTCTGCGGTTCAGCGTACAggg                                                                                                                                                                                                                                                     >  1:104496/1‑300 (MQ=255)`  
                                                       `ttcgtACATTGAGGAATACTTAGGTGACTTACGGTCCCTTGAAAATCTCCAAGAGGCTATCGTCAAGATGTCCATGATTAGCTCTAAGGTTATCTGCTTAGTGAATCCTGCTGGTATCACCCAGCCACGCCGACTGACCAAAGCTCAGACTGGTGACTTCGTTACTGGTCGTCCAGAAGACATCTCGTTCATCCAACTGGAGAAGCAAGCAGACTTTACTGTAGCTAAAGCCGTAAGTGACGTTATCGAGGCTCGCCTTTCGTTTGCCTTTATGTTGAACTCTGCGGTTCAGCGGACAggg                                                                                                                                                                                                                                                     >  1:76935/1‑300 (MQ=255)`  
                                                       `ttcgtACATTGAGGAATACTTAGGTGACTTACGGTCCCTTGAAAATCTCCAAGAGGCTATCGTCAAGATGTCCATGATTAGCTCTAAGGTTATCGGCTTAGTGAATCCTGCTGGTATCACCCAGCCACGCCGACTGACCAAAGCTCAGACTGGTGACTTCGTTACTGGTCGTCCAGAAGACATCTCGTTCCTCCAACTGGCGAAGCAAGCAGACTTTACTGTAGCTAAAGCCGTAAGTGACGTTATCGAGGCTCGCCTTTCGTTGGCATTTATGTTGACCACTGCGGTTCAGCGtctatgg                                                                                                                                                                                                                                                     >  1:382686/1‑295 (MQ=255)`  
                                                           `tACATTGAGGAATACTTAGGTGACTTACGGTCCCTTGAAAATCTCCAAGAGGCTATCGTCAAGATGTCCATGATTAGCTCTAAGGTTATCGGCTTAGTGAATCCTGCTGGTATCACCCAGCCACGCCGACTGACCAAAGCTCAGACTGGTGACTTCGTTACTGGTCGTCCAGAAGACATCTCGTTCCTCCAACTGGAGAAGCAAGCAGACTTTACTGTAGCTAAAGCCGTAAGTGACGTTATCGAGGCTCGCCTTTCGTTTGCCTTTATGTTGAACTCTGCGGTTCAGCGTACAGGTgctt                                                                                                                                                                                                                                                 >  1:351214/1‑298 (MQ=255)`  
                                                            `aacaggAGGAATACTGAGGTGACTTAAGGGCCCTTGAAAATCTCCAAGAGGCTATCGTCAAGATGTCCATGATTAGCTCTAAGGTTATCGGCTTAGTGAATCCTGCTGGTATCACCCAGCCACGCCGACTGACCAAAGCTCAGACTGGTGACGTTGTTACTGGTCGTCCAGAAGACATCTCGTTCCTCCAACTGGAGAAGCAAGCAGACCTTACTGTAGCTAAAGCCTTAAGTGACTTTATCGAGGCTCGCCTTTCGTTTGCCTTTATGTTGAACGCTGCGGTTCAGCGTACAGGTGAACg                                                                                                                                                                                                                                                <  1:497744/296‑1 (MQ=255)`  
                                                                 `gAGGAATACTTAGGTGACTTACGGTCCCTTGAAAATCTCCAAGAGGCTATCGTCAAGATGTCCATGATTAGCTCTAAGGTTATCGGCTTAGTGAATCCTGCTGGTATCACCCAGCCACGCCGACTGACCAAAGCTCAGACTGGTGACTTCGTTACTGGTCTTCCAGAAGACATCTCGTTCCTCCAACTGGAGAAGCAAGCAGACTTTACTGTAGCTAAAGCCGTAAGTGACGTTATCGAGGCTCGCCTTTCGTTTGCCTTTATGTTGAACTCTGCGGTTCAGCTTACAGGTGAACGTTTGa                                                                                                                                                                                                                                           >  1:387560/1‑301 (MQ=255)`  
                                                                 `gAGGAATACTTAGGTGACTTACGGTCCCTTGAAAATCTCCAAGAGGCTATCGTCAAGATGTCCATGATTAGCTCTAAGGTTATCGGCTTAGTGAATCCTGCTGGTATCACCCAGCCACGCCGACTGACCAAAGCTCAGACTGGTGACTTCGTTACTGGTCGTCCAGAAGACATCTCGTTCCTCCAACTGGAGAAGCAAGCAGACTTTACTGTAGCTAAAGCCGTAAGTGACGTTATCGAGGCTCGCCTTTCGTTTGCCTTTATGTTGAACTCTGCGGTTCAGCGTACAGGTGAACGTGTGa                                                                                                                                                                                                                                           >  1:425678/1‑301 (MQ=255)`  
                                                                      `aTACTTAGGTGACTTACGGTCCCTTGAAAATCTCCAAGAGGCTATCGTCAAGATGTCCATGATTAGCTCTAAGGTTATCGGCTTAGTGAATCCTGCTGGTATCACCCAGCCACGCCGACTGACCAAAGCTCAGACTGGTGACTTCGTTACTGGTCGTCCAGAAGACATCTCGTTCCTCCAACTGGAGAAGCAAGCAGACCTTACTGTAGCTAAAGCCGTAAGTGACGTTATCGAGGCTCGCCTTTCGTTTGCCTTTATGTTGAACTCTGCGGTTCAGCGTACAGGTGAACGTGTGAccgcc                                                                                                                                                                                                                                      >  1:212797/1‑301 (MQ=255)`  
                                                                      `aTACTTAGGTGACTTACGGTCCCTTGAAAATCTCCAAGAGGCTATCGTCAAGATGTCCATGATTAGCTCTAAGGTTATCGGCTTAGTGAATCCTGCTGGTATCACCCAGCCACGCCGACTGACCAAAACTCAGACTGGTGACTTCGTTACTGGTCGTCCAGAAGACATCTCGTTCCTCCAACTGGAGAAGCAAGCAGACTTTACTGTAGCTAAAGCCGTAAGTGACGTTATCGAGGCTCGCCTTTCGTTTGCCTTTATGTTGAAGTCTGCGACTCAGGGTACAGGTGAACTTGTGAccgcc                                                                                                                                                                                                                                      >  1:67917/1‑301 (MQ=255)`  
                                                                         `cTTAGGTGACTTACGGTCCCTTGAAAATCTCCAAGAGGCTATCGTCAAGATGTCCATGATTAGCTCTAAGGTTATCGGCTTAGTGAATCCTGCTGGTATCACCCAGCCACGCCGACTGACAAAAGCTCAGACTGGTGACTTCGTTACTGGTCGTCCAGAAGACATCTCGTTCCTCCAACTGGAGAAGCAAGCAGACTTTACTGTAGCTAAAGCCGTAAGTGACGTTATCGAGGCTCGCCTTTCGTTTGCCTTTATGTTGAACTCTGCGGTTCAGCGTAcctgtctcttatacacatctccc                                                                                                                                                                                                                                   >  1:153158/1‑279 (MQ=255)`  
                                                                            `gctcggagatgtgtataagagacagctcCAAGAGGCTATCGTCAAGATGTCCATGATTAGCTCTAAGGTTATCGGCTTAGTGAATCCTGCTGGTATCACCCAGCCACGCCGACTGACCAAAGCTCAGACTGGTGACTTCGTTACTGGTCGTCCAGAAGACATCTCGTTCCTCCAACTGGAGAAGCAAGCAGACTTTACTGTAGCTAAAGCCGTAAGTGACGTTATCGAGGCTCGCCTTTCGTTTGCCTTTATGTTGAACTCTGCGGTTCAGCGTACAGGTGAACGTGTGACCGCCGAagag                                                                                                                                                                                                                                <  1:3951/276‑1 (MQ=255)`  
                                                                                `gACTTACGGTCCCTTGAAAATCTCCAAGAGGCTATCGTCAAGATGTCCATGATTAGCTCTAAGGTTATCGGCTTAGTGAATCCTGCTGGTATCACCCAGCCACGCCGACTGACCAAAGCTCAGACTGGTGACTTCGTTACTGGTCGTCCAGAAGACATCTCGTTCCTCCAACTGGAGAAGCAAGCAGACTTTACTGTAGCTAAAGCCGTAAGTGACGTTATCGAGGCTCGCCGTTCGTTTGCCTTTATGTTGAACTCTGCGGTTCAGCGTACAGGTGAACGTGTGACCGCCGAAGAGACTc                                                                                                                                                                                                                            >  1:367323/1‑301 (MQ=255)`  
                                                                                  `cTTACGGTCCCTTGAAAATCTCCAAGAGGCTATCGTCAAGATGTCCATGATTAGCTCTAAGGTTATCGGCTTAGTGAATCCTGCTGGTATCACCCAGCCACGCCGACTGACCAAAGCTCAGACTGGTGACTTCGTTACTGGTCGTCCAGAAGACATCTCGTTCATCCAACTGGAGAAGCAAGCAGACTTTACTGTAGCTAAAGCCGTAAGTGACGTTATCGAGGCTCGCCTTTCGTTTGCCTTTATGTTGAACTCTGCGGTTCAGCGTACAGGTGAACGTGTGACCGCCGAAGAGATTCgg                                                                                                                                                                                                                          >  1:399156/1‑301 (MQ=255)`  
                                                                                          `cccTTGAACATCTCCAAGAGGCTATCGTCAAGATGTCAATGATTAGCTCTAAGTTTATCGGCTTAGTGAACCCTGTGGGTATCACCAAGCCACGCCGACTGACCAAAGCTCAGACTGGTGACTTCGTTACTGGTCGTCCAGAAGACATCTCGTTCCTCCAACTGGAGAAGCAAGCAGACTTTACTGTAGCTAAAGCCGTAAGTGACGTTATCGAGGCTCGCCTTTCGTTTGCCTTTATGTTGAACTCTGAGGTTCAGCGTACAGGTGAACGTGTGACCGCCGAAGAGATTCGGTATGTAGc                                                                                                                                                                                                                  <  1:114323/301‑1 (MQ=255)`  
                                                                                          `cccTTGAAAATCTCCAAGAGGCTATCGTCAAGATGTCCATGATTAGCTCTAAGGTTATCGGCTTAGTGAATCCTGCTGGTATCACCCATCCACGCCGACTGACCAAAGCTCAGACTGGTGACTTCGTTACTTGTCGTCCAGAAGACATCTCGTTCCTCAAACTGGAGAATCAAGCAGACTTTACTGTAGCTCAAGCCGTAAGTGACGTTATCTAGGCTCGCCTTTCGTTTGCCTTTATGTTGAACTCTGCGGTTCAGCGTACAGTTGAACGTGTGACCGCCGAAGAGATTCGGTATGTAGc                                                                                                                                                                                                                  >  1:43446/1‑301 (MQ=255)`  
                                                                                          `cccTTGAAAATCTCCAAGAGGCTATCGTCAAGATGTCCATGATTAGCTCTAAGGTTATCGGCTTAGTGAATCCTGCTGGTATCACCCAGCCACGCCGACTGACCAAAGCTCAGACTGGTGACTTCGTTACTGGTCGTCCAGAAGACATCTCGTTCCTCCAACTGGAGAAGCAAGCAGACTTTACTGTAGCTAAAGCCGTAAGTGACGTTATCGAGGCTCGCCTTTCGTTTGCCTTTATGTTGAACTCTGCGGTTCAGCGTACAGGTGAACGTGTGACCGCCGAAGAGATTCGGTATGTAGc                                                                                                                                                                                                                  >  1:306650/1‑301 (MQ=255)`  
                                                                                                   `ttctcCAAGAGGCTATCGTCAAGATGTCCATGATTAGCTCTAAGGTTATCGGCTTAGTGAATCCTGCTGGTATCACCAAGCCACGCCGACTGACCAAAGCTCAGACTGGTGACTTCGTTACTGGTCGTCCAGAAGACATCTCGTTCCTCCAACTGGAGAAGCAAGCAGACTTTACTGTAGCTAAAGCCGTAAGTGACGTTATCGAGGCTCGCCTTTCGTTTGCCTTTATGTTGAACTCTGCGGTTCAGCGTACAGGTGAACGTGTGACCGCCGAAGAGATTCGGTATGTAGCTTCTGAACt                                                                                                                                                                                                         <  1:354894/300‑1 (MQ=255)`  
                                                                                                   `aTCTCCAAGAGGCTATCGTCAAGATGTCCATGATTAGCTCTAAGGTTATCGGCTTAGTGAATCCTGCTGGTATCACCCAGCCACGCCGACTTACCAAAGCTCAGACTGGTGACTTCGTTACTGGTCGTCCAGAAGACATCTCGTTCCTCCAACTGGAGAAGCAAGCAGACTTTACTGTAGCTAAAGCCGTAAGTGACGTTATCGAGGCTCGCCTTTCGTTTGCCTTTATGTTGAACTCTGCGGTTCAGCGTACAGGTGAACGTGTGACCGCCGAAGAGATTCGGTATGTAGCTTCTGAACt                                                                                                                                                                                                         >  1:320321/1‑301 (MQ=255)`  
                                                                                                     `ctcCAAGAGGCTATCGTCAAGATGTCCATGATTAGCTCTAAGGTTATCGGCTTAGTGAATCCTGCTGGTATCACCCAGCCACGCCGACTGACCAAAGCTCAGACTGGTGACTTCGTTACTGGTCGTCCACAAGACATCTCGTTCCTCCACCTGGAGACGCAAGCAGACTTTCCTGTAGCTAAAGCCGTAAGTGACGTTATCGAGGCTCGCCTTTCGTTTGCCTTTATGTTTAACTCTGCGGTTCAGCGTACAGTTGCACGTGTGACCGCCGAAGAGATTCTGTCTGTAGCTTTTGAACtgt                                                                                                                                                                                                       >  1:371888/1‑299 (MQ=255)`  
                                                                                                          `agagGCTATCGTCAAGATGTCCATGATTAGCTCTAAGGTTATCGGCTTAGTGAATCCTGCGGGTATCACCAAGCCACGCCGACTGACCAAAGCTCAGACTGGTGACTTCGTTACTGGTCGTCCAGAAGACATCTCGTTCCTCCAACTGGAGAAGCAAGCATACTTTACTGTTGCTAAAGCCGTAAGTGACGTTATCGAGGCTCGCCTTTCGTTTGCCTTTATGTTGAACTCTGCGGTTCAGCGTACAGGTGAACGTGTGACCGCCGAAGAGATTCGGTATGTAGCTTCTGAACTTGAAGAt                                                                                                                                                                                                  <  1:403640/301‑1 (MQ=255)`  
                                                                                                              `gCTATCGTCAAGATGTCCATGATTAGCTCTAAGGTTATCGGCTTAGTGAATCCTGCTGGTATCACCCAGCCACGCCGACTGACCAAAGCTCAGACTGGTGACTTCGTTACTGGTCGTCCAGAAGACATCTCGTTCCTCCAACTGGAGAAGCAAGCAGACTTTACTGTAGCTAAAGCCGTAAGTGACGTTATCGAGGCTCGCCTTTCGTTTGCCTTTATGTTGAACTCTGCGGTTCAGCGTACAGGTGAACGTGTGACCGCCGAAGAGATTCGGTATGTAGCTTCTTAACTTGCAGCTACtt                                                                                                                                                                                              >  1:273217/1‑301 (MQ=255)`  
                                                                                                                   `ggTCAAGATGTCCATGATTATCTCTAAGGTTATCGGCTTAGTGAATCCTGCTGGTATCACCCAGCCACGCCGACTGACCAAAGCTCAGACTGGTGACTTCGTTACTGGTCGTCCAGAAGACATCTCGTTCCTCCAACTGGAGAAGCAAGCAGACTTTACTGTAGCTAAAGCCGTAAGTGACGTTATCGAGGCTCGCCTTTCGTTTGCCTTTATATTGAACTCTGCGGTTCAGCGTACAGGTGAACGTGTGACCGCCGAAGAGATTCGGTATGTAGCTTCTGAACTTGAAGATACTTTAggt                                                                                                                                                                                         <  1:187505/300‑1 (MQ=255)`  
                                                                                                                   `cttCAAGATTTCCATGTTTAGCTCTAAGGTTATCGTCCTAGTGTATCCTGCTGGTTTCACCCAGTCACGCCGACTTACCAACGCTCAGCCTTGTGACTTCGTTACTGGTCGTCCATAAGACATCTCGTTCCTCCAACTCTAGAAGCAAGCATACTTTACTGTAGCTAAAGCCTTAAGTGACGTTATCGAGGCTCGCCTTTCGTTTGCCTTTATGTTGAACTCTGCGGTTCAGCGTACAGGTGAACGTGTGACCGCCGAAGAGATTCGGTATGTAGCTTCTGAACTTGAAGATACTTTAggt                                                                                                                                                                                         <  1:6315/299‑1 (MQ=255)`  
                                                                                                                   `cGTCAAGATGTCCATGATTAGCTCTAAGTTTATCGGCTTAGTGAATCCTGCTGGTATCACCCAGCCACGCCGACTGACCAAAGCTCAGACTGGTGACTTCGTTACTGGTCGTCCAGAAGACATCTCGTTCCTCCAACTGGAGAAGCAAGCAGACTTTACTGTAGCTAAAGCCGTAAGTGACGTTATCGAGGCTCGCCTTTCGTTTGCCTTTATGTTGAACTCTGCGGTTCAGCGTACAGGTGAACGTGTGACCGCCGAAGAGATTCGGTATGTAGCTTCTGAACTTGAAGAAACTTTAggt                                                                                                                                                                                         <  1:83061/301‑1 (MQ=255)`  
                                                                                                                    `gTCAAGATGTCCATGATTAGCTCTAAGGTTATCGGCTTAGTGAATCCTGCTGGTATCACCCAGCCACGCCGACTGACCAAAGCTCAGACTGGTGACTTCGTTACTGGTCGTCCAGAAGACATCTCGTTCCTCCAACTGGAGAAGCAAGCAGACTTTACTGTAGCTAAAGCCGTAAGTGACGTTATCGAGGCTCGCCTTTCGTTTGCCTTTATGTTGAACTCTGCGGTTCAGCGTACAGGTGAACGTGTGACCGCCGAAGAGATTCGGTATGTAGCTTCTGAACTTGAAGATACTTTAggtg                                                                                                                                                                                        >  1:486086/1‑301 (MQ=255)`  
                                                                                                                    `gTCAAGATGTCCATGATTAGCTCTAAGGTTATCGGCTTAGTGAATCCTGCTGGTATCACCCAGCCACGCCGACTGACCAAAGCTCAGACTGGTGACTTCGTTACTGGTCGTCCAGAAGACATCTCGTTCCTCCAACTGGAGAAGCAAGCAGACTTTACTGTAGCTAAAGCCGTAAGTGACGTTATCGAGGCTCGCCTTTCGTTTGCCTTTATGTTGAAATCTGCGGTTCAGCGTACAGGTGAACGTGTGACCGACGAAGAGATTCGGTATGTAGCTTCTGAACTTGAAGATCCTTTAggtt                                                                                                                                                                                        >  1:124358/1‑300 (MQ=255)`  
                                                                                                                       `aaGATGTCCATGATTAGCTCTAAGGTTATCGGCTTAGTGAATCCTGCTGGTATCACCCAGCCACGCCGACTTACCAAAGCTCAGACTGGTGACTTCGTTACTTGTCGTCCAGAAGACATCTCGTTCCTCCAACTGGAGAAGCAAGCAGACTTTACTGTAGCTAAAGCCGTAAGTGACGTTATCGAGGCTCGCCTTTCGTTTGCCTTTATGTTGAACTCTGCGGTTCAGCGTACAGGTGAACGTGTGACCGCCGAAGAGATTCGGTATGTAGCTTCTGAACTTGAAGATACTTTAGgtgg‑tg                                                                                                                                                                                    <  1:1090/301‑1 (MQ=255)`  
                                                                                                                             `tCCATGATTAGCTCTAAGGTTATCGGCTTAGTGAATCCTGCTGGTATCACCCAGCCACGCCGACTGACCAAAGCTCAGACTGGTGACTTCGTTACTGGTCGTCCAGAAGACATCTCGTTCCTCCAACTTGAGAAGCAAGCAGACTTTACTGTAGCTAAAGCCGTAAGTGACGTTATCGAGGCTCGCCTTTCGTTTGCCTTTATGTTGAACTCTGCGGTTCAGCGTACAGGTGAACGTGTTACCGCCGAAGAGATTCGGTATGTAGCTTCTGCACTTGATGATACTTTAGGTGG‑TGTCTAct                                                                                                                                                                              >  1:195929/1‑301 (MQ=255)`  
                                                                                                                              `tgtataagagacagaatgTTTTCGGCTTAGTGAATCCTGCTGGTTTCACCCAGCCACGCCGCCTGACCAAAGCTCAGACTGGTGACTTCGTTACTGGTCGTCCAGAAGACATCTCGTTCCTCCAACTGGAGAAGCAAGCAGACTTTACTGTAGCTAAAGCCGTAAGTGACGTTATCGAGGCTCGCCTTTCGTTTGCCTTTATGTTGAACTCTGCGGTTCAGCGTACAGGTGAACGTGTGACCGCCGAAGAGATTCGGTATGTAGCTTCTGAACTTGAAGATACTTTAGGTGG‑TGTCTActc                                                                                                                                                                             <  1:416690/284‑1 (MQ=255)`  
                                                                                                                              `taagagacaGCTCTAAGGTTATCGGCTTAGTGAATCCTGCTGGTATCACCCAGCCACGCCGACTGCCCAAAGCTCAGACTGGTGACTTCGTTACTGGTCGTCCAGAAGACATCTCGTTCCTCCAACTGGAGAAGCAAGCAGACTTTACTGTAGCTAAAGCCGTAAGTGACGTTATCGAGGCTCGCCTTTCGTTTGCCTTTATGTTGAACTCTGCGGTTCAGCGTACAGGTGAACGTGTGACCGCCGAAGAGATTCGGTATGTAGCTTCTGAACTTGAAGATACTTTAGGTGG‑TGTCTActc                                                                                                                                                                             <  1:467454/293‑1 (MQ=255)`  
                                                                                                                              `ccATGATTAGCTCTAAGGTTATCGGCTTAGTGAATCCTGCTGGTATCACCCAGCCACGCTGACTGACCAAAGCTCAGACTGGTGACTTCGTTACTGGTCGTCCAGAAGACATCTCGTTCCTCCAACTGGAGAAGCAAGCAGACTTTACTGTAGCTAAAGCCGTAAGTGACGTTATCGAGGCTCGCCTTTCGTTTGCCTTTATGTTGAACTATGCGGTTCAGCGTACAGGTGAACGTGTGACCGCCGAAGAGATTCGGTATGTAGCTTCTGAACTTGAAGATACTTTAGGTGG‑TGTCTActc                                                                                                                                                                             <  1:48142/301‑1 (MQ=255)`  
                                                                                                                              `cagtGATTAGCTCTAAGGTTATCGGCTTAGTGAATCCTGCTGGTATCACCCAGCCACGCCGACTGACCAAAGCTCAGACTGGTGACTTCGTTACTGGTCGTCCAGAAGACATCTCGTTCCTCCAACTGGAGAAGCAAGCAGACTTTACTGTAGCTAAAGCCGTAAGTGACGTTATCGAGGCTCGCCTTTCGTTTGCCTTTATGTTGAACTCTGCGGTTCAGCGTACAGGTGAACGTGTGACCGCCGAAGAGATTAGGTATGTAGCTTCTGAACTTGAAGATACTTTAGGTGG‑GGTCTActc                                                                                                                                                                             <  1:269099/298‑1 (MQ=255)`  
                                                                                                                              `aagagacaggCTCAAAGGTTATCGGCTTAGTGAATCCTGCTGGTATCACCCAGCCACGCCGACTGACCAAAGCTCAGACTGGTGAATTCGTGACTGGTCGTCCAGAAGACATCTCGTTCCTCCAACTGAAGAAGCAAGCAGACTTTACTGTAGCTAAAGCCGTAAGTGACGTTATCGAGGCTCGCCTTTCGTTTGACTTTATGTTGAACTCTGCGGTTCAGCGTACAGGTGAACGTGTGACCGTCGAAGAGATTCGGTATGTAGCTTCTGAACTTGAAGATACTTTAGGTGG‑TGTCTActc                                                                                                                                                                             <  1:58162/292‑1 (MQ=255)`  
                                                                                                                                 `tGATTTGCTCTAAGGTTATCGGCTTAGTGAATCCTGCTGGTATCACCCATCCACGCCGACTGACCAAAGCTCAGACTTGTGACTTCGTTACTGGTCGTCCAGAAGACATCTCGTTCCTCAAACTGGAGAAGCAAGCAGACTTTACTGTAGCTAAAGCCGTACGTGACGTTATCGAGGCTCGCCTTTCGTTTGCCTTTATGTTGAACTCTGCGGTTCAGCGTACAGGAGAACGTGTGACCGCCGAAGAGATTCTGTATGTAGCTTCTGAACTTGAAGATACTTTAGGTGGCTGTCtcttata                                                                                                                                                                           >  1:506979/1‑295 (MQ=255)`  
                                                                                                                                  `gATTAGCTCTAAGGTTATCGGCTTAGTGAATCCTGCTGGTATCACCCAGCCACGCCGACTGACCAAAGCTCAGACTGGTGACTTCGTTACTGGTCGTCCAGAAGACATCTCGTTCCTCCAACTGGAGAAGCAAGCAGACTTTACTGTAGCTAACGCCGTAAGTGACGTTATCGAGGCTCGCCTTTCGTTTGCCTTTATGTTGAACTCTGCGATTCAGCGTACAGGTGAACGTGTGACCGCCGAAGAGATTCGGTATGTAGCTTCTGAACTTGAAGATACTTTAggtatgtctcttatacaa                                                                                                                                                                          >  1:33050/1‑286 (MQ=255)`  
                                                                                                                                   `aTTAGCTCTAAGTTTATCTGCTTATTGAGACCTGCTGGTATCACCCAGCCACGCCGACTGTCGAAAGCTCAGACTGTTTACTTCGGTACTGATCGTCCATAAGACATCTCGTTCCTCCAACTGGATAAGCTAGCAGACTTTACTGTAGCTAAAGCCGTAAGTGACGTTATCTAGGCTCGCCTTTCGTTTGCCTTTATGTTTAACTCTGCTGTTCATCGTACCGTTGAACTTGTGACCGCCGAAGAGATTCGGTATTTAGCTTCTTAACTTTAAGATACTTTAGGTTG‑TGTCTACTCTATcc                                                                                                                                                                        <  1:259628/301‑1 (MQ=255)`  
                                                                                                                                       `gCTCTAAGGTTATCGGCTTAGTGAATCCTGCTGGTATCACCCAGCCACGCCGACTGACCAAAGCTCAGACTGGTGACTTCGTTACTGGTCGTCCAGAAGACATCTCGTTCCTCCAACTGGAGAAGCAAGCAGACTTTACTGTAGCTAAAGCCGTAAGTTACGTTATCGAGGCTCGCCTTTCGTTTGCCTTTATGTTGAACTCTGCGGTTCAGCGTACAGGTGAACGTGTGACTGCCGAAGAGATTCGGTATGTAGCTTCTGAACTTGAAGATACTTTAGGTGG‑TGTCTACTCTATCCtttt                                                                                                                                                                    >  1:461334/1‑300 (MQ=255)`  
                                                                                                                                       `gCTCTAAGGTTATCGGCTTAGTGAATCCTGCTGGTATCACCCAGCCACGCCGACTGACCAAAGCTCAGACTGGTGACTTCGTTACTGGTCGTCCAGAAGACATCTCGTTCCTCCAACTGGAGAAGCAAGCAGACTTTACTGTAGCTAAAGCCGTAAGTGACGTTATCGAGGCTCGCCTTTCGTTTGCCTTTATGTTGAACTCTGCGGTTCAGCGTACAGGTGAACGTGTGACCGCCGAAGAGATTCGGTATGTAGCTTCTGCACTTGAAGATACTTTAGGTGG‑TGTCTACTATATCCtttt                                                                                                                                                                    >  1:128263/1‑300 (MQ=255)`  
                                                                                                                                       `gCTCTAAGGTTATCGGCTTAGTGAATCCTGCTGGTATCACCCAGCCACGCCGACTGACCAAAGCTCAGACTGGTGACTTCGTTACTGGTCGTCCAGAAGACATCCCGTTCCTCCAACTGGAGAAGCAAGCAGACTTTACTGTAGCTAAAGCCGTAAGTGACGTTATCGAGGCTAGCCTTTCGTTTGCCTTTATGTTGAACTCTGCGGTTCAGCGTACAGGTGAACGTGTGACCGCCGAAGAGATTCGGTATGTAGCTTCTGAACTTGAAGATACTTTAGGTGG‑TGTATACTCTATctgtct                                                                                                                                                                    >  1:335335/1‑296 (MQ=255)`  
                                                                                                                                       `agcgactggTTATCGGCTGATTTCATCCTGCTTGGATCACCCTGCCACGCCGACTGACCCAAGCTCAGACTGGTGACTTCCTTACTGTTCGTCCCGATGCCATCTCGTTCCTCCACCTCGAGTAGCAAGCAGACTTTACTGTCGCTAAAGCTGTAAGTGTGGTTTTCGAGGCTCGGCTTTCGTTTGCTTTTTTGTTGAACTCTGCGGTTCATCGTACAGTTGAACGTGTTACCACCGAAGAGATTCGGTATGTAGCTTCTGAACTTGAAGATACTTTAGGTGG‑TGTCTACTCTATCCTTtc                                                                                                                                                                    <  1:45280/294‑1 (MQ=255)`  
                                                                                                                                        `ctctAAGGTTATCGGCTTAGTGAATCCTGCTGGTATCACCCAGCCACGCTGACTGACCAAAGCTCAGACTGGTGACTTCGTTACTGGTCGTCCAGAAGACATCTCGTTCCTCCAACTGGAGAAGCAAGCAGACGTTACTGTAGCTAAAGCCGTAAGTGACGTTATCGAGGCTCGCCTTTCGTTTGCCTTTATGTTGAACTCTGCGGTTCAGCGTACAGGTGAACGTGTGACCGCCGAAGAGATTCGGTATGTAGCTTCTGAACTTGAAGATACTTTAGGTGG‑TGTCTACTCTATCCTTtct                                                                                                                                                                   <  1:457508/301‑1 (MQ=255)`  
                                                                                                                                              `ggTTATCGGCTTAGTGAATCCTGCTGGTATCACCCAGCCACGCCGACTGACCAAAGCTCAGACTGGTGACTTCGTTACTGGTCGTCCATAAGACATCTCGTTCCTCCAACTGGAGATGCAAGCATACTTTACTGTAGCTACAGCCGTAAGTGACGTTATCGAGGCTCGCCTTTCGTTTGCCTTTATGTTGAACTCTGCGGTTCAGCGTACATGTGAACGTGTGACCGCCGAAGATATTCGGTATGTAGCTTCTGAACTTGAAGATACTTTCGGTGG‑TGTCTActcctttctcttatacaca                                                                                                                                                             >  1:476793/1‑285 (MQ=255)`  
                                                                                                                                               `gTTATCGGCTTAGTGAATCCTGCTGGTATCACCCAGCCACGCCGACTGACCAAAGCTCAGACTGGTGACTTCGTTACTGGTCGTCCAGAAGACATCTCGTTCCTCCAACTGGAGAAGCAAGCAGACTTTACTGTAGCTAAAGCCGTAAGTGACGTTATCGAGGCTCGCCTTTCGTTTGCCTTTATGTTGAACTCTGCGGTTCAGCGTACAGGTGAACGTGTGACCGCCGAAGAGATTCGGTATGTAGCTTCTGAACTTGAAGATACTTTAGGTGG‑GGTCTActcctgtctcttatacacat                                                                                                                                                            >  1:408851/1‑284 (MQ=255)`  
                                                                                                                                                      `gCTTAGTGAATCCTGCTGGTATCACCCAGCCACGCCGACTGACCAAAGCTCAGACTGGTGACTTTGTTACTGGTCGTCCAGAAGACATCTCGTTCCTCCAACTGGAGACGCAAGCAGACTTTACTGTAGCTAAAGCCGTAAGTGACGTTATCGAGGCTCGCCTTTCGTTTGCCTTTCTGTTGAACTCTGCGGTTCAGCGTACAGGTGAACGTTTGTCCGCCGAACAGATTCGGTATGTAGCTTCTGAACTTGAGAATACTTTAGGTGG‑TGTCTACTCTATCTTTTCTCAAGAATTACAAtt                                                                                                                                                     >  1:301825/1‑301 (MQ=255)`  
                                                                                                                                                         `tAGTGAATCCTGCTGGTATCACCAAGCAACGCCGACTGACCAAAGCTCAGACTGGTGACATAGTTACTAGTCGTCCAGACGACATCTCGCTCCTCTAACTGGAGAAGCAAGCAGACTTTACTGTAGATAAAGCCGTAAGTGACGTTATCGAGGCTCGCCTTTCGATTGACTTTATGTTGTAGTCTGCGGTTCAGCGTACAGGTGAACGTGTGACCGCCGAAGAGATTCGGTATGTCGCTTATGAATTTGATGATACTTTAGGTGG‑TGTCTACTCTATCCGTTCTCAAGAATTACAATTGcc                                                                                                                                                  >  1:169267/1‑301 (MQ=255)`  
                                                                                                                                                           `gTGAATCCTGCTGGTATCACCCAGCCACGCCGACTGACCAAAGCTCAGACTGGTGACTTCGTTACTGGTCGTCCAGAAGACATCTCGTTCCTCCAACTGGAGAAGCAAGCAGACTTTACTGTAGCTAAAGCCGTAAGTGACGTTATCGAGGCTCGCCTTTCGTTTGCCTTTATGTTGAACTCTGAGGTTCAGCGTACAGGTGAACGTGTGACCGCCGAAGAGATTCGGTATGTAGCTTCTGAACTTGAAGATACTTTAGGTGG‑TGTCTACTCTATCCTTTCTCAAGAATTACAATTGCctc                                                                                                                                                >  1:100028/1‑301 (MQ=255)`  
                                                                                                                                                               `aTCCTGCTGGTATCACCCAGCCACGCCGACTGACCAAAGCTCAGACTGGTGACTTCGTTACTGGTCGTCCAGAAGACATCTCGTTCCTCCAACTGGAGAAGCAAGCAGACTTTACTGTAGCTAAAGCCGTAAGTGACGTTATCGAGGCTCGCCTTTCGTTTTCCTTTATGTTGAACTCTGCGGTTCAGCGTACAGGTGACCGTGTGACCTCCGAAGAGATTCGGTATGTAACTTCTGAACTTGAAGATACTTTAGTTGT‑TGTCTACTCTATCCTTTCTCAAGCATTACAATTTCctccttt                                                                                                                                            >  1:132178/1‑297 (MQ=255)`  
                                                                                                                                                                `tCCTGCTGGTATCACCCAGCCACGCCGACTGACCAAAGCTCAGACTGGTGACTTCGTTACTGGTCGTCCAGAAGACATCTCGTTCCTCCAACTGGAGAAGCAAGCAGACTTTACTGTAGCTAAAGCCGTAAGTGACGTTATCGAGGCTCGCCTTTCGTTTGCCTTTATGTTGAACTCTGCGGTTCAGCGTACAGGTGAACGTGTGACCGCCGAAGAGATTCGGTATGTAGCTTCTGAACTTGAAGATACTTTAGGTGG‑TGTCTACTCTATCCTTTCTCAAGAATTACAATTGCCTCTGGTa                                                                                                                                           <  1:164894/301‑1 (MQ=255)`  
                                                                                                                                                                       `ggTATCACCCAGCCACGCCGACTGACCAAAGCTCAGACTGGTGACTTCGTTACTGGTCGTCCAGAAGACATCTCGTTCCTCCAACTGGAGAAGCAAGCAGACTTTACTGTAGCTAAAGCCGTAAGTGACGTTATCGAGGCTCGCCTTTCGTTTGCCTTTATGTTGAACTCTGCGGTTCAGCGTACAGGTGAACGTGTGACCGCCTAAGAGATTCGGTATGTAGCTTCTGAACTTGAAGATACTTTAGGTGG‑TGTCTACTCTATCCTTTCTCAAGAATTACAATTGCCTCTGGTACGAGTGc                                                                                                                                    <  1:442803/301‑1 (MQ=255)`  
                                                                                                                                                                               `ccAGCCACGCCGACTGACCAAAGCTCAGACGGGTGACGTCGTTACTGGTCGTCCAGAAGACATCTCGTTCCTCCAACTGGAGAAGCAAGCAGACTTTACTGTAGCTAAAGCCGTAAGTGACGTTATCGAGGCTCGCCTTTCGTTTGCCTTTATGTTGAACTCTGCGGTTCAGCGTACAGGTGAACGTGTGACCGCCGAAGAGATTCGGTATGTAGCTTCTGAACTTGAAGATACTTTAGGTGG‑TGTCTACTCTATCCTTTCTCAAGAATTACAATTGCCTCTGGTACGAGTGCTCTTGAAg                                                                                                                            <  1:263839/301‑1 (MQ=255)`  
                                                                                                                                                                                `tgtataagagacaggACCAAAGCTCAGACTGGTGACTTCGTTACTGGTCGTCCAGAAGACATCTCGTTCCTCCAACTGGAGAAGCAAGCAGACTTTACTGTAGCTAAAGCCGTAAGTGACGTTATCGAGGCTCGCCTTTCGTTTGCCTTTATGTTGAACTCTGTGGTTCAGCGTACAGGTGAACGTGTGACCGCCGAAGAGATTCGGTATGTAGCTTCTGAACTTGACGATACTTTAGGTGG‑TGTCTACTCTATCCTTTCTCAAGAATTACAATTGCCTCTGGTACGAGTGCTCTTGAAGc                                                                                                                           <  1:236669/287‑1 (MQ=255)`  
                                                                                                                                                                                           `ttataagagacagcAGACTGGTGAATTCGTTACTGGTCGTCCAGAAGACATCTCGTTCCTCCAACTGGAGAAGCAAGCAGACTTTACTGTAGCTAAAGCCGTAAGTGACGTTATCGAGGCTCGCCTTTCGTTTGCCTTTATGTTGAACTCTGCGGTTCAGCGTACAGGTGAACGTGTGACCGCCGAAGAGATTCGGTATGTAGCTTCTGAACTTGAAGATACTTTAGGTGG‑TGTCTACTCTATCCTTTCTCAAGAATTACAATTGCCTCTGGTACGAGTGCTCTTGAAGCAACTACAAGcc                                                                                                                <  1:359358/288‑1 (MQ=255)`  
                                                                                                                                                                                           `aCTGTCCACAGCTCGACCTGTTGACTTCGTTACTGTTCGTCCAGACGCCATCTCGTGCCTCCAACTGCAGAAGCAAGCTGACTTTACTGTAGCTAAAGCCGTAAGTGACGTTATCGAGGCTCGCCTTTCGTTTGCCTTTAGGTTGAACTCTGCGGTTCAGCGTACAGGTGAACGTGTGACCGCCGAAGAGATTCGGTCTGTAGCTTCTGAACTTGAAGATACTTTAGGTGG‑TGTCTACTCTATCCTTTCTCAAGAATTACTATTGCCTCTGGTACGAGTGCTCTTGAAGCAACTACAAGcc                                                                                                                <  1:135872/301‑1 (MQ=255)`  
                                                                                                                                                                                                     `gCTCAGATTGGTGACTTCGTTACTGGTCGTCCAGAAGACATCTCGTTCCTCCAACTGGAGAAGCAAGCAGACTTTACTGTAGCTAAAGCCGTAAGTGACGTTATCGAGGCTCGCCTTTCGTTTGCCTTTATGTTGAACTCTGCGGTTCAGCGTACAGGTGAACGTGTGACCGCCGAAGAGATTAGGTATGTAGCTTCTTAACTTGAAGATACTTTAGGTGG‑TGTCTACTCTATCCTTTCTCAAGAATTACAATTTCCTCTGGTACGAGTGCTTTTGAAGCAACTACAAGCCACGCAACAGa                                                                                                      >  1:92757/1‑301 (MQ=255)`  
                                                                                                                                                                                                          `gACTGGTGACTTCGTTACTGGTCGTCCAGAAGACATCTCGTTCCTCCAACTGGAGAAGCAAGCAGACTTTACTGTAGCTAAAGCCGTAAGTGACGTTATCGAGGCTCGCCTTTCGTTTGCCTTTATGTTGAACTCTGCGGTTCAGCGTACAGGTGAACGTGTGACCGCCGAAGAGATTCGGTATGTAGCTTCTGAACTTGAAGATACTTTAGGTGG‑TGTCTACTCTATCCTTTCTCAAGAATTACAATTGCCTCTGGTACGAGTGCTCTTGAAGCAACTACAAGcctgtctcttatacaca                                                                                                 >  1:178908/1‑286 (MQ=255)`  
                                                                                                                                                                                                             `aagagacagcGTTACTGGTCGTCCAGAAGACATCTCGTTCCTCCAACTGGAGAAGCAAGCAGACTTTACTGTAGATAAAGCCGTAAGTGACGTTATCGAGGCTCGCCTTTCGTTTGCCTTTATGTTGAACTCTGCGGTTCAGCGTACAGGTGAACGTGTGACCGCCGAAGAGATTAGGTATGTAGCTTCTGAACTTGAAGATACTTTAGGTGG‑TGTCTACTCTATCCTTTCTCAAGAATTACAATTGCCTCTGGTACGAGTGCTCTTGAAGCAACTACAAGCCACGCAACAGATTCCTGAg                                                                                              <  1:430301/292‑1 (MQ=255)`  
                                                                                                                                                                                                                   `cTTTGTTACTGGTCGTCCAGAAGACATCTCGTTCCTCCAACTGGAGAAGCAAGCAGACTTTACTGTAGCTAAAGCCGTAAGTGACGTTATCGAGGCTCGCCTTTCGTTTGCCTTTATGTTGAACTCTGCGGTTCAGCGTACAGGTGAACGTGTGACCGCCGAAGAGATTCGGTATGTAGCTTCTGAACTTGAAGATACTTTAGGTGG‑TGTCTACTCTATCCTTTCTCAAGAATTACAATTGCCTCTGGTACGAGTGCTCTTGAAGCAACTACAAGCCACGCAACAGATTCCTGAGTTACCt                                                                                        >  1:446677/1‑301 (MQ=255)`  
                                                                                                                                                                                                                      `cGTTACTGGTCGTCCAGAAGACATCTCGTTCCTCCAACTGGAGAAGCAAGCAGACTTTACTGTAGCTAAAGCCGTAAGTGACGTTATCGAGGCTCGCCTTTCGTTTGCCTTTATGTTGAACTCTGCGGTTCAGCGTACAGGTGAACGTGTGACCGCCGAAGAGATTCGGTATGTAGCTTCTGAACTTGAAGATACTTTAGGTGG‑TGTCTACTCTATCCTTTCTCAAGAATTACAATTGCCTCTGGTACGAGTGCTCTTGAAGCAACTACAAGCCACGCAACAGATTTCTGAGTTACCTAAg                                                                                     >  1:20929/1‑301 (MQ=255)`  
                                                                                                                                                                                                                      `cGTTACTGGTCGTCCAGAAGACATCTCGTTCCTCAAACTGGAGAAGCAAGCAGACTTTACTGTAGCTAAAGCCGTAAGTGACGTTATCGAGGCTCGCCTTTCGTTTGCCTTTATGTTGAACTCTGCGGATCAGCGTACAGGTGAACGTGTGACCGCCGAAGAGATTCGGAATGTAGCTACTGAATTTGAAGATACTTTAGGTGG‑TGTCTACTCTATCCTTTCTCAAGAATTACTATTGCTTCTGGTACGAGTGCTCTTGAAGCAAATACAAGACACGAAACCGATTCCTGAGTTAACTAAg                                                                                     >  1:55396/1‑301 (MQ=255)`  
                                                                                                                                                                                                                       `gTTACTGGTCGTCCAGAAGACATCTCGTTCCTCCAACTGGAGAAGCAAGCAGACTTTACTGTAGCTAAAGCCGTAAGTGACGTTATCGAGGCTCGCCTTTCGTTTGCCTTTATGTTGAACTCTGCGGTTCAGCGTACAGGTGAACGTGTGACCGCCGAAGAGATTCGGTATGTAGCTTCTGAACTTGCAGATACTTTAGGTGG‑TGTATACTCTATCCTTTCTCAAGAATTACAATTTCCCCTGTACCGATTACTCTTGAAGCACTTACAATCCACCCAACAgcgtctcctcttacccactt                                                                                    >  1:491375/1‑282 (MQ=255)`  
                                                                                                                                                                                                                        `ttACTGGTCGTCCAGAAGACATCCCGGTCCTCCAACTGGAGAAGCAAGCAGACTTTACTGTAGCTAAAGCCGTAAGTGACGTTATCGAGGCTCGCCTTTCGTTTGCCTTTATGTTGAACTCTGCGGTTCAGAGTACAGGTGAACGTGTGACCGCCGAAGAGATTCGGTATGTAGCTTCTGAACTTGAAGATACTTTAGGTGG‑TGTCTACTCTATCCTTTCTCAAGAATTACAATTGCCTCTGGTACGAGTGCTCTTGAAGCAACTACAAGCCACGCAACAGATTCCTGAGTTACCTAAGGa                                                                                   <  1:148900/301‑1 (MQ=255)`  
                                                                                                                                                                                                                         `gttggaggaacgagaagaCATCTCGTTCCTCCAACTGGAGAAGCAAGCAGACTTTACTGTAGCTAAAGCCGTAAGTGACGTTATCGAGGCTCGCCTTTCGTTTGCCTTTATGTTGAACTCTGCGGTTCAGCGTACAGGTGAACGTGTGGCCTCCGAAGAGATTTGGTATGTAGCTTCTGAACTTTAATATACTTTAGGTGG‑TGTCTACTCTATCCTTTCTCAAGAATTACAATTGCCTCTGGTACGAGTGCTCTTGAAGCAACTACAAGCCACGCAACAGATTCCTGAGTTACCTAAGGaa                                                                                  <  1:326614/289‑1 (MQ=255)`  
                                                                                                                                                                                                                                      `gaagaCATCTCGTTCCTCCAACTGGAGAAGCAAGCAGACTTTACTGTAGCTAAAGCCGTAAGTGACGTTATCGATGCTCGCCTTTCGTTTGCCTTTATGTTGAACTCTGCGGTTCAGCGTACAGGTGAACGTGTGACCGCCGAAGAGATTCGGTATGTAGCTTCTGAACTTGAAGATACTTTAGGTGG‑TGTCTACTCTATCCTTTCTCAAGAATTACAATTGCCTCTGGTACGAGTGCTCTTGAAGCAACTACAAGCCACGCAACAGATTCCTGAGTTACCTAAGGAAGCCGTAGAGCCaa                                                                     >  1:310744/1‑301 (MQ=255)`  
                                                                                                                                                                                                                                      `gaagaCATCTCGTTCCTCCAACTGGAGAAGCAAGCAGACTTTACTGTAGCTAAAGCCGTAAGTGACGTTATCGAGGCTCGCCTTTCGTTTGCCTTTATGTTGAACTCTGCGGTTCAGCGTACAGGTGAACTTGTGACCGCCGAAGAGATTCGGTATGTAGCTTCTGAACTTGAAGATACTTTAGTTGG‑TGTATACTCTATCCTTTCCCAAGAATTACAATTGCCTCTGGTACTAATGCTCTTGAAGAAACTACAAGCCACGCACCAGATTCCTGAGTTACCTAAGTCAGCGGTAATGCCaa                                                                     >  1:316960/1‑301 (MQ=255)`  
                                                                                                                                                                                                                                         `gaCATCTCGTTCCTCCAACTGGAGAAGCAAGCAGACTTTACTGTAGCTAAAGCCGTAAGTGACGTTATCGAGGCTCGCCTTTCGTTTGCCTTTATGTTGAACTCTGCGGTTCAGCGTACAGGTGAACGTGTGACCGCCGAAGAGATTCGGTATGTAGCTTCTGAACTTGAAGATACTTTAGGTGG‑TGTCTACTCTATCCTTTCTCAAGAATTACAATTGCCTCTGGTACGAGTGCTCTTGAAGCAACTACAAGCCACGCAACAGATTCCTGAGTTACCTAAGGAAGCCGTAACGCCAACCa                                                                  >  1:200270/1‑301 (MQ=255)`  
                                                                                                                                                                                                                                               `taagatacagaaCTGGAGAAGCAAGCAGACTTTACGGTAGCTAAAGCCGTAAGTGACGTTATCTAGGCTCGCCTTTCGTTTGTCTGTATGTTGAACTCTGCGGTTCAGTGTACAGGTTAACGTGTTACCGCCGAAGAGATTCGGTATGTAGCTTCTGAACTTGAAGATACTTTAGGTGG‑TGTCTACTCTATCCTTTCTCAAGAATTACAATTGCCTCTGGTACGAGTGCTCTTGAAGCAACTACAAGCCACGCAACAGATTCCTGAGTTACCTAAGGAAGCCGTAGAGCCAACCATTAGTa                                                            <  1:256167/291‑1 (MQ=255)`  
                                                                                                                                                                                                                                                `taagagacagaCTGGAGAAGCAAGCAGACTTTACTGTAGCTAAAGCCGTAAGTGACGTTATAGAGGCTCGCCTTTCGTTTGCCTTTATGTTGAACTCTGCAGTTCAGCGTACAGGTGAACGTGTGACCGCCGAAGAGATTCGGTATGTAGCTTCTGAACTTGAAGATACTTTAGGTGG‑TGTCTACTCTATCCTTTCTCAAGAATTACAATTGCCTCTGGTACGAGTGCTCTTGAAGCAACTACAAGCCACGCAACAGATTCCTGAGTTACCTAAGGAAGCCGTAGAGCCAACCATTAGTAc                                                           <  1:471456/291‑1 (MQ=255)`  
                                                                                                                                                                                                                                                `cGTTCCTCCAACTGTAGAAGCAAGCAGACTTTACTGTAGCTAATGCCGTAAGTGACGTTATCGAGGCTCGCCTTTCGTTTGCCTTTATGTTTAACTCTGCGGTTCAGCGTACAGGTGAACGTGTGACCGCCGAAGAGATTCGGTATGTAGCTTCTGAACTTGAAGATACGTTAGGTGG‑TGTCTACTCTATCCTTTCTCAAGAATTACAATTGCCTCTGGTACGAGTGCTCTTGAAGCAACTACAAGCCACGCAACAGATTCCTGAGTTACCTAAGGAAGCCGTAGAGCCAACCATTAGTAc                                                           <  1:217214/301‑1 (MQ=255)`  
                                                                                                                                                                                                                                                  `ttCCTCCAACTGGAGAAGCAAGCAGACTTTACTGTAGCTAAAGCCGTAAGTGACGTTATCGAGGCTCGCCTTTCGTTTGCCTTTATGTTGAACTCTGCGGTTCAGCGTACAGGTGAACGTGTGACCGCCGAAGAGATTCGGTATGTAGCTTCTGAACTTGAAGATACTTTAGGTGG‑TGTCTACTCTATCCTTTCTCAAGAATTACAATTGCCTCTGGTACGAGTGCTCTTGTAGCAACTACAAGCCACGCAACAGATTCCTGAGTTACCTAAGGAAGCCGTAGAGCCAACCATTAGTAcct                                                         >  1:204278/1‑299 (MQ=255)`  
                                                                                                                                                                                                                                                   `tcctccAACTGGAGAAGCAAGCAGACTTTACTGTAGCTAAAGCCGTAAGTGACGTTATCGAGGCTCGCCTTTCGTTTGCCTTTATGTTGAACTCTGCTGTTCAGCGTACAGGTGAACGTGTGACCGCCGAAGAGATTCGGTATGTAGCTTCTGAACTTGAAGAGACTTTAGGTGG‑TGTCTACTCTATCCTTTCTCAAGAATTACAATTGCCTCTGGTACGAGTGCTCTTGAAGCAACTACAAGCCACGCAACAGATTCCTGAGTTACCTAAGGAAGCCGTAGAGCCAACCATTAGTACAgg                                                        <  1:232062/301‑1 (MQ=255)`  
                                                                                                                                                                                                                                                    `cctccAACTGGAAAAGCAAGCAGACTTTACTGTAGCTAAAGCCGTAAGTGACGTTATCGAGGCTCGCCTTGCAATTGCCTTTATTTTGAACTCTGCGGCTCAGCTTACAGGTGAACCTGTGACCGCCGAAGAGACTCGGTATGTAGCTTCTGAACTTGAACATACTTTAGGTGG‑TGTCTACTCTATCCTTTCTCAAGAATTACAATTGCCTCTGTTACGAGTGCTCTTGAAGCAACTACATGCCACGCTACAGATTCCTGCGTTACCTAAGTCAGTCGTCGATCCACCTATCAGTAccagt                                                       >  1:246137/1‑297 (MQ=255)`  
                                                                                                                                                                                                                                                     `ctccATCTGGAGAAGCAAGCAGACTTTACGGTAGCTAAAGCCGTAAGTGACGTTATCGAGGCTCGCCTTTCGTTTGCCTTTATGTTGAACTCTGCGGTTCAGCGTACAGGTGAACGTGTGACCGCCGAAGAGATTCGGTATGTAGCTTCTGAACTTGAAGATACTTTAGGTGG‑TGTCTACTCTATCCTTTCTCAAGAATTACAATTGCCTCTGGTACGAGTGCTCTTGAAGCAACTACAATCCACGCAACAGATTCCTGAGTTACCTAAGGAAGCCGTAGAGCCAACCATTAGTACAGGTc                                                      <  1:174735/301‑1 (MQ=255)`  
                                                                                                                                                                                                                                                     `ctccAACTGGAGAAGCAAGCAGACTTTACTGTAGCTAAAGCCGTAAGTGACGTTATCGAGGCTCGCCTTTCGTTTGCCTTTATGTTGAACTCTGCGGTTCAGCGTACAGGTGAACGTGTGACCGCCGAAGAGATTCGGTATGTAGCTTCTGAACTTGAAGATACTTTAGGTGG‑TGTCTACTCTATCCTTTCTCAAGAATTACAATTGCCTCTGGTACGAGTGCTCTTGAAGCAACTACAAGCCACGCAACAGATTCCTGAGTTACCTAAGGAAGCCGTAGAGCCAACCATTAGTACAGGTc                                                      <  1:119362/301‑1 (MQ=255)`  
                                                                                                                                                                                                                                                         `aaCTGGAGAAGCAAGCAGACTTTACTGTAGCTAAAGCCGTAAGTGACGTTATCGAGGCTCGCCTTTCGTTTGCCTTTATGTTGAACTCTGCGGTTCAGCGTACAGGTGAACGTGTGACCGCCGAAGAGATTCGGTATGTAGCTTCTGAACTTGAAGATACTTTAGGTGG‑TGTCTACTCTATCCTTTCTCAAGAATTACAATTGCCTCTGGTACGAGTGCTCTTGAAGCAACTACAAGCCACGCAACAGATTCCTGAGTTACCTAAGGAAGCCGTGGAGCCAACCCTTAGTACAGGTCTGGa                                                  >  1:391619/1‑301 (MQ=255)`  
                                                                                                                                                                                                                                                                `gaAGCAAGCAGACTTTACTGTAGCTAAAGCCGTAAGTGACGTTATCGAGGCTCGCCTTTCGTTTGCCTTTATGTTGAACTCTGCGGTTCAGCGTACAGGTGAACGTGTGACCGCCGAAGAGATTCGGTATGTAGCTTCTGAACTTGAAGATACTTTAGGTGG‑TGTCTACTCTATCCTTTCTCAAGAATTACAATTGCCTCTGGTACGAGTGCTCTTGAAGCACCTACAAGCCACGCAACAGATTCCTGAGTTACCTAAGGAAGCCGTAGAGCCAACCATTATTACAGGTCTGGCAGCAAtt                                           >  1:363174/1‑301 (MQ=255)`  
                                                                                                                                                                                                                                                                  `agcaagcaGACTTTACTGTAGCTAAAGCCGTAAGTGACGTTATCGAGGCTCGCCTTTCGTTTGCCTTTATGTTGAACTCTGCGGTTCAGCGTACAGGTGAACGTGTGACCGCCGAAGATATTCGGTATGTAGCTTCTGAACTTGAAGATACTTTAGGTGG‑TGTCTACTCTATCCTTTCTCAAGAATTACAATTGCCTCTGGTACGAGTGCTCTTGAAGCAACTACAAGCCACGCACCAGATTCCTTAGTTACCTAAGGAAGCCGTAGAGCCAACCATTAGTACAGGTATGGAAGCAATTgg                                         >  1:281437/1‑301 (MQ=255)`  
                                                                                                                                                                                                                                                                    `aaagcaGGCTTTACTGTAGCTAAAGCCGTAAGTGACGTTATCGAGGCTCGCCTTTCGTTTGCCTGTAGGTTGAACTCTGCGGTTCAGCGTACAGGTGAACGTGTGACCGCCGAAGAGATTCGGTATGTAGCTTCTGAACTTGAAGATACTTTAGGTGG‑TGTCTACTCTATCCTTTCTCAAGAATTACAATTGCCTCTGGTACGAGTGCTCTTGAAGCAACTACAAGCCACGCAACAGATTCCTGAGTTACCTAAGGAAGCCGTAGAGCCAACCATTAGTACAGGTCTGGAAGCAATTGGTc                                       <  1:341268/300‑1 (MQ=255)`  
                                                                                                                                                                                                                                                                            `cTTTACTGTAGCTAAAGCCGTAAGTGACGTTATCGAGGCTCGCCTTTCGTTTGCCTTTATGTTGAACTCTGCGGTTCAGCGTACAGGTGAACGTGTGACCGCCGAAGAGATTCGGTATGTAGCTTCTGAACTTGAAGATACTTTAGGTGG‑TGTCTACTCTATCCTTTCTCAAGAATTACAATTGCCTCTGGTACGAGTGCTCTTGAAGCAACTACAAGCCACGCAACAGATTCCTGAGTTACCTAAAGAAGCCGTAGCGACAACCATTAGTACAGGTCTGGAAAAAATTAGTCGAGGACaa                               >  1:87214/1‑301 (MQ=255)`  
                                                                                                                                                                                                                                                                                       `cTAAAGCCGTAAGTGACGTGATAGAGGCTCGCCTTTCGTTTGCCTTTATGTTGAACTCTGCGGTTCAGCGTACAGGTGAACGTGTGACCGCCGAAGAGATTCGGTATGTAGCTTCTGAACTTGAAGATACTTTAGGTGG‑TGTCTACTATATCATTTCTCAAGAATTACAATTGCCTCTGGTACGAGTGCTCTTGAAGAAACTACAAGCCACGCAACAGATTCCTGAGTTACCTAAGGAAGCCGTAGAGCCAACCATTAGTACAGGTCTGGAAGCAATTGGTCGAGGACAAGACCTTGATaa                    <  1:29685/301‑1 (MQ=255)`  
                                                                                                                                                                                                                                                                                                   `gTGACGTCATCGAGGCTAGTCGTTTGTGTGCCTATATGTAGAACAATGCGGTTCAGCGTACAGGTAAACGTGTGACCGCAGAAGAGATTCGGGATGTAGCTTCTGAACTTTAAAATACTTTAGGTGG‑TGGCTACTCTATCCTTTCTCAAGAATTACAATTGCCTCTGGTACGAGTGCTCTTGAAGCAACTACAAGCCACGCAACAGATTCCTGAGTTACCTAAGGAAGCCGTAGAGCCAAACATTAGTACAGGTCTGGAAGCAATTGGTCGAGGACAAGACCTTGATAAGCTGGAGCGgtg        <  1:502760/301‑1 (MQ=255)`  
                                                                                                                                                                                                                                                                                                      `aCGTTATCGAGTCTCGCCTTTCGTTTGCCTTTATGTTGAACTCTGCGGTTCAGCGTACAGGTGAACGTTTGACCGCCGAAGAGATTCGGTATGTAGCTTCTGAACTTGAAGATACTTTAGGTTG‑TGTCTACTCTATCCTTTCTCAAGTATTACAATTGCCTCTGGTACGAGTGCTCTTGAAGCAACTACAAGCCACGCAACAGATTCCTGAGTTACCTAAGGAAGCCGTAGAGCCAACCATTAGTACCGGTCTGGAAGCAATTGGTCGAGGACAAGACCTTGATAAGCTGGAGCGgtgtgt     <  1:25105/301‑1 (MQ=255)`  

                                                                                                                                                                                                                                                                                                         `|`                                                                                                                                                                                                                                                                                                               
`CTTGCCCATACATCCCGATTCGGATGGTCAGACTAGATGGTGAATCCTACGGTCGTTCGTACATTGAGGAATACTTAGGTGACTTACGGTCCCTTGAAAATCTCCAAGAGGCTATCGTCAAGATGTCCATGATTAGCTCTAAGGTTATCGGCTTAGTGAATCCTGCTGGTATCACCCAGCCACGCCGACTGACCAAAGCTCAGACTGGTGACTTCGTTACTGGTCGTCCAGAAGACATCTCGTTCCTCCAACTGGAGAAGCAAGCAGACTTTACTGTAGCTAAAGCCGTAAGTGACGCTATCGAGGCTCGCCTTTCGTTTGCCTTTATGTTGAACTCTGCGGTTCAGCGTACAGGTGAACGTGTGACCGCCGAAGAGATTCGGTATGTAGCTTCTGAACTTGAAGATACTTTAGGTGG‑TGTCTACTCTATCCTTTCTCAAGAATTACAATTGCCTCTGGTACGAGTGCTCTTGAAGCAACTACAAGCCACGCAACAGATTCCTGAGTTACCTAAGGAAGCCGTAGAGCCAACCATTAGTACAGGTCTGGAAGCAATTGGTCGAGGACAAGACCTTGATAAGCTGGAGCGGTGTGTCAC  >  NC_001604.1/20982‑21579`

  

Alignment Legend

Aligned base mismatch/match (shaded by quality score): `ATCG/ATCG` `< 3 ≤` `ATCG/ATCG` `< 10 ≤` `ATCG/ATCG` `< 19 ≤` `ATCG/ATCG` `< 34 ≤` `ATCG/ATCG` `< 38 ≤` `ATCG/ATCG`

Unaligned base: `atcg`    Masked matching base: `atcg`    Alignment gap: `‑`    Deleted base: `‑`
